# Appendicitis in Children

**Published:** 1905-11-11

**Authors:** D'Arcy Power

**Affiliations:** Surgeon to St. Bartholomew's Hospital and to the Bolingbroke Hospital. Senior Surgeon to the Victoria Hospital for Children, Chelsea


### Hospital Clinics.

### APPENDICITIS IN CHILDREN.

By D'Arcy Power, F.R.C.S.Eng., Surgeon to St. Bartholomew's Hospital and to the Bolingbroke Hospital. Senior Surgeon to the Victoria Hospital for Children, Chelsea.

(A Clinical Lecture delivered at St. Bartholomew's Hospital, November 1, 1905.)

(*Continued from page* 84.)

*Prognosis.*—Most of the pitfalls attending the diagnosis and treatment of appendicitis in adults are present in cases of appendicitis in children. Some, perhaps, in an exaggerated form, because adults complain and form their own conclusions, whilst the ailments of children are often overlooked or misinterpreted. A rectal examination should be made in every case where the diagnosis is at all doubtful, and I would again lay especial stress on the fact that the pulse gives a better clue to the child's condition in cases of appendicitis than the temperature or even the physical signs.

A child with appendicitis, therefore, needs the most careful and continual watching. A falling temperature with cessation of pain, even when the child sleeps, regains some colour and asks for food, is not of good import if the pulse remains as quick as it was before the apparent improvement. It means that the appendix has given way and has either discharged a concretion or has poured its contents into the abdominal cavity; sometimes it has become gangrenous. Children do not suffer from toxæmia as quickly as adults, and so long as they are kept warm and are free from pain they remain happy, for even in the worst cases of appendicitis a child may not seem extremely ill to a superficial observer and may even deceive an anxious mother. But the surgeon should not be deceived. Centuries of experience have taught him the truth of the aphorism laid down by Hippocrates, ὁ δὲ καιρὸς ὀξύς, "occasio præceps," "the occasion is fleeting" during which remedies may be successfully applied. The previous condition of the little patient and the continued acceleration of the pulse should have warned him that appearances are fallacious, and should have taught him to guard against the peritonitis and collapse which are imminent by an early operation. Sometimes I am told that such an one cannot have had appendicitis, for the bowels have been regularly open throughout his illness. Constipation is not necessarily associated with inflammation of the appendix. Diarrhœa occurs in a certain proportion of cases and is perhaps of bad omen, whilst the second case I told you about ended fatally though the bowels remained regular to the end. The abdominal wall is generally rigid and motionless over an inflamed appendix, but in children the belly may feel quite supple and the respiratory rhythm may remain unaffected, even when there is general septic peritonitis. The absence of rigidity is, I think, most marked when the suppurating appendix is deeply placed in the abdomen with coils of small intestine adherent round it and cutting off the inflammatory products from direct communication with the parietal layer of the peritoneum.

*Treatment.*—You have often heard me say that in cases of septic peritonitis the prognosis is bad, but that it is better in children than in women, and in women than in men. It follows therefore that every case should be given the chance of recovery afforded by an operation. Some of you will remember the condition of the girl who is now in Paget Ward suffering from the effects of a general peritonitis which began with an inflamed appendix. She was almost moribund when we had her in the theatre, but in spite of this I hastily opened her abdomen in the middle line and in either loin putting in large drainage tubes. My house surgeon, Mr. G. H. Colt, worked hard to save her life and spent many hours in superintending the subcutaneous infusion of saline infusion. Within a few days she had received twenty-nine pints in this manner, and although she was desperately ill, she is now in a fair way to recover. Early operation in the majority of cases would save us such ventures, but early operation means necessarily early diagnosis on the part of the medical practitioner under whose notice the patient first comes, for neither in hospital nor in private practice does a surgeon see a case of appendicitis in its first stage. It is imperative, therefore, that you should know the early symptoms and be able to differentiate them from the other and more common affections of childhood.

*Differential diagnosis.*—The differential diagnosis is of the greatest importance in the case of children with appendicitis. It is calculated that only about 15 per cent. of all the cases of appendicitis occur in children below the age of puberty, so that when I am summoned to an obscure abdominal case in a child I think rather of intussusception or of obstruction by a band than of appendicitis. If peritonitis is already present I try to eliminate the tuberculous and pneumococcic forms before I accuse the appendix. The pain may lead to a diagnosis of renal colic, or the limping may direct attention so entirely to the right hip as to cause the abdominal condition to be overlooked in the earlier stages of the affection. On the other hand, I have seen a psoas abscess treated as a case of appendicitis, nor was the mistake so gross as it might appear, for the disease must have been limited to the front of the bodies of the vertebræ, as there was never any deformity or tenderness over the child's spine. Simple constipation with fæcal impaction may be mistaken for a case of appendicitis, but this is a less deadly error than to treat a patient with appendicitis as though he were suffering from fæcal impaction, because in the latter case a medical man thinks that he has performed his whole duty as soon as he has emptied the lower bowel.

Finally, in some rare cases the later stages of an enteritis in a child may be mistaken for the obstruction due to inflammation of the appendix.

Intussusception begins much more acutely than appendicitis. It is not accompanied by any rise of temperature in the early stages and the child quickly becomes collapsed. Examination of the abdomen does not show any particular rigidity or swelling in the right iliac fossa, and in many cases of intussusception there is an absence of the usual resistance on the right side because the invagination has drawn the cæcum upwards. When a swelling can be felt in an intussusception it is more movable than in a case of appendicitis, more elongated, more easily felt at one time than another owing to the peristalsis of the intestinal wall, and it is generally situated somewhere in the course of the colon. In appendicitis the tumour is fixed at or near the right iliac fossa, it is tender, and it may be visible through the abdominal walls when the muscles are relaxed. The abdominal walls themselves may be œdematous when there is an appendix abscess, but I have never seen them œdematous in intussusception, even in those rare cases where the intestine has been gangrenous. A rectal examination in a case of intussusception may prove that the apex of the swelling is within easy reach of the finger, and is in the lumen of the bowel, whilst in appendicitis the swelling is limited to the right side of the pelvis and is as evidently outside the intestine. In intussusception the examining finger may be covered with blood-stained mucus, whilst in appendicitis the bowel often contains fæces, or if it be empty in the later stages the sphincter has relaxed and the rectum may be ballooned.

Obstruction due to strangulation by a band has a sudden and definite onset like intussusception, which distinguishes it from appendicitis. The pain is greater than in appendicitis, and is limited to a single spot or is referred to the region of the umbilicus. At first there is no rise of temperature, and, as soon as the large intestine has been emptied, the constipation is so absolute that neither fæces nor flatus is passed. Vomiting as a rule is a marked feature, but the local rigidity and tenderness characteristic of appendicitis are absent. The course run by a case of strangulation by a band is extremely insidious and may be so gentle that a child is often thought to be suffering only from obstinate constipation. The appearances improve as the pain decreases with the onset of gangrene and the little patient may die of peritonitis at the very moment the doctor is assuring the mother that her child is getting better. Such a disastrous prognosis would be less frequent if everyone made it a rule to count the pulse daily, and if necessary to examine the child's abdomen under an anæsthetic, in all cases of painful constipation which have begun suddenly.

It is not difficult as a rule to distinguish between peritonitis associated with inflammation of the appendix, and that due to the presence of tubercle bacilli or pneumococci. In tuberculous peritonitis there is usually a long history of illness; the patient is wasted, suffers from diarrhœa, hectic fever, and has night sweats. Pneumococcic peritonitis is rarely primary or single. The child has had pneumonia and on examination the other serous cavities of the body are found to be affected.

But both in tubercle and pneumococcic infection the signs sometimes point only to acute intestinal obstruction. A timely exploration of the abdomen will alone yield a correct diagnosis and such an early exploration is fortunately the best method of treating each form of peritonitis.

Appendicitis and renal colic are sometimes mistaken for each other. Stone in the kidney is not uncommon in children, but it is not a frequent cause of pain, partly because the kidneys in children seem to tolerate large calculi, especially if they are embedded in the cortex; partly because the majority of renal calculi are very small and are soon passed into the bladder. Hæmaturia is a very constant symptom of renal calculus in children and the loss of blood is often sufficient to make the children very anæmic; on the other hand, the urine may remain quite clear and a microscopic examination will be required to show the presence of crystals of oxalate of lime which reveal the cause of the symptoms. In every doubtful case a skiagraph should be taken because the picture tells more in children than in adults. A few months ago I removed a stone from each kidney in a child with the most satisfactory results. The patient had been an invalid for a long time on account of obscure abdominal pain. A skiagraph was taken and in each kidney a stone was seen to be present as large as a brace-button.

It is somewhat more difficult to distinguish between tuberculous pyonephrosis and appendicitis. A careful examination shows that the swelling lies higher in the abdomen than is the case in appendicitis, whilst in nearly every case of pyonephrosis the urine affords some indication of the condition of the kidney. The pain is not a safe guide, because the genito-crural nerve may be irritated equally in pyonephrosis, in renal colic, and in appendicitis, so that in each form of inflammation the right testicle may be retracted and the pain may be referred to the inner side of the right thigh.

When the history is obscure and the child is only seen in the later stages some cases of enteritis may be mistaken for appendicitis because the diarrhœa and pain are replaced by constipation and apathy. The explanation lies, I suppose, in the fact that during the early stages of intestinal inflammation the mucous membrane alone is involved and there is painful diarrhœa. The muscular and serous coats afterwards become inflamed and the bowel is paralysed so that the diarrhœa gives place to absolute constipation. The absorption of toxin leads to the listless appearance of the child and to the vomiting which continues to the end and becomes stercoraceous.

From what you have heard to-day, gentlemen, I think you will agree with me that the subject of appendicitis in children deserves the time we have devoted to its consideration. The symptoms it presents and the course it runs do not always render it easy to recognise. A larger number of cases occur every year and it behoves us therefore to be increasingly on the alert because we cannot hope for a correct and rational treatment unless we make an early and accurate diagnosis.

